# The Effects of Heat Treatment Temperatures on the Properties of 316L Stainless Steel Produced via Laser Powder Bed Fusion

**DOI:** 10.3390/ma18133167

**Published:** 2025-07-03

**Authors:** Yizhi Zhou, Mingxia Chai, Fu Zheng, Zhiyong Li

**Affiliations:** 1 College of Systems & Society, The Australian National University, Canberra, ACT 2600, Australia; u7850365@anu.edu.au; 2 School of Mechanical Engineering, Shandong University of Technology, Zi’bo 255049, China; cmx110617@163.com (M.C.); 18645161688@163.com (F.Z.)

**Keywords:** laser powder bed fusion (PBF-LB/M), heat treatment, microstructure, mechanical properties, 316L stainless steel

## Abstract

316L stainless steel (316L SS) exhibits excellent corrosion resistance, mechanical properties, and biocompatibility, but the rapid melting and solidification of the laser powder bed fusion (PBF-LB/M) process reduce the properties of the newly formed parts. This study aims to enhance the mechanical properties of PBF-LB/M PBF-LB/M-formed 316L SS parts by investigating the effects of various heat treatment temperatures. The results show that an appropriate heat treatment temperature can improve the microstructure and mechanical properties of the formed parts. Lower temperatures have minimal effects on performance; however, at 1100 °C, recrystallization occurs, resulting in more uniform grain structures, improved densification, and substantial stress relief. The residual stress is reduced by 85.59% compared to the untreated PBF-LB/M samples, while the ferrite content is significantly decreased, making the phase structure more homogeneous. Although both yield strength and tensile strength decrease, plasticity improves by 21.11%.

## 1. Introduction

316L SS is a low-carbon austenitic stainless steel with excellent corrosion resistance, biocompatibility, and weldability, and it is one of the most widely used high-performance stainless steels, especially for harsh corrosive environments and when high standards of cleanliness are required [1,2]. 316L SS powder has become one of the commonly used materials in PBF-LB/M metal additive manufacturing, with high material utilization, which allows for the preparation of complex parts and components for personalized manufacturing [3,4]. However, the rapid melting and solidification of the additive manufacturing process results in high residual stresses in the formed parts [5], which will be prone to fatigue failure and crack initiation [6]. Furthermore, insufficient melting of the powder particles and poor interlayer metallurgical bonding result in reduced densification of the part [7,8], while the high temperature gradient leads to tissue inhomogeneity, which makes the workpieces manufactured via the PBF-LB/M process very different from the traditionally machined workpieces in terms of structure and performance, largely limiting their direct applications in industry [9].

Therefore, enhancing the performance of PBF-LB/M-fabricated parts has become a critical research direction. Suitable process parameters can effectively improve the mechanical properties of PBF-LB/M parts [10]. For example, laser power, scanning speed, and laser spot diameter affect the geometrical characteristics of the scanning trajectory and quality of the formed parts [11,12], while suitable scanning spacing can avoid unmelted defects, and reducing the exposure time can inhibit splash spheroidization [13]. The laser scanning strategy also has important influence on the surface quality, residual stress, and deformation of the molten metal [14]. However, manufacturing defects cannot be completely eliminated by adjusting the processing parameters. Shi et al. found that heat treatment reduced the strength of PBF-LB/M-formed 316L SS but increased its ductility due to reductions in the dislocation density and cellular microstructures [15]. Heat treatment also changes the internal phase composition of the PBF-LB/M-fabricated specimens, which reduces the microhardness of the specimens [16], as well as the dissolution of the internal subgrains of the specimens, which has an important effect on their mechanical strength [17]. Heat treatment at 1200 °C for one hour significantly reduced the porosity and cracks in the joint area of the W and 316L SS of the W/316L SS specimens [18], and Tascioglu et al. [19] showed that heat treatment under furnace-cooled conditions reduced the porosity of 316L SS specimens. However, Morozova et al. [20] examined heat treatment under air-cooled or water-cooled conditions, which had an unfavorable effect on the porosity of 316L SS specimens, and the study by Ronneberg et al. [21] showed that heat treatment did not affect the porosity of 316L SS specimens. Therefore, further research on the effect of heat treatment on the porosity of additively manufactured 316L SS samples is still required. The stress-relieving heat treatment reduced the yield strength of PBF-LB/M-formed 316L SS parts to a higher level than that of hot-rolled parts without significantly affecting the ultimate strength and elongation of the specimens [22]. The strength properties of PBF-LB/M-fabricated 316L SS parts were not significantly altered by the heat treatment at 800 °C, but the ductility was significantly reduced due to the precipitation of the internal embrittled σ phase. After treatment at 1100 °C, the strength and ductility of the specimens decreased significantly [23]. Heat treatment can improve the corrosion resistance of the PBF-LB/M-formed 316L SS samples by changing the structure of the specimens, making the subgrain boundaries migrate and disappear [24], homogenizing the internal non-equilibrium structure, and increasing the oxide content in the passive film [25,26]. However, it has also been shown that heat-treated additively manufactured 316L SS specimens form MnS inclusions, leading to a drastic decrease in pitting resistance [27].

As a common post-treatment, heat treatment can significantly improve the internal microstructure and composition of the material, leading to improvements in properties such as strength, porosity, and microhardness. However, most previous studies focus on limited performance parameters. With a single-factor test, through an in-depth analysis of the heat treatment temperature on the quality of mechanisms used to form the PBF-LB/M channel specimens, the microstructure of the annealed heat-treated specimens was analyzed at different temperatures, residual stresses, densifications, phase compositions, and hardnesses to examine the changes in mechanical properties and reveal the mechanisms behind the impact of heat treatment on the quality of the PBF-LB/M specimens, which is of great significance to the practical industrial application of 316L products produced via PBF-LB/M.

## 2. Materials and Methods

### 2.1. Materials and Manufacturing

**Figure 1:**
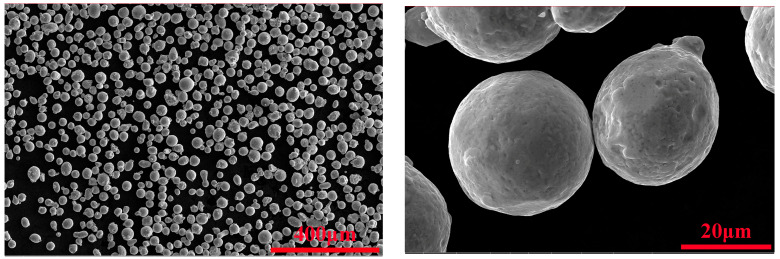
SEM morphology of 316L stainless steel powder.

The experimental material is 316L SS powder produced by Jiangsu Weilali New Material Technology Co., Ltd. (Xuzhou, China). The results of the powder composition analysis are shown in Table 1. The particle size of the powder was detected using an X-ray diffraction analyzer (D8 ADVANCE, Karlsruhe, Germany), and the measured particle size range was 15–53 μm, with a normal distribution of D10 = 19.8 μm, D50 = 30.3 μm, and D90 = 45.0 μm. The powder was observed using a scanning electron microscope, and it had a well-characterized spherical shape with a relatively uniform distribution of particle sizes, as shown in Figure 1.

**Figure 2:**
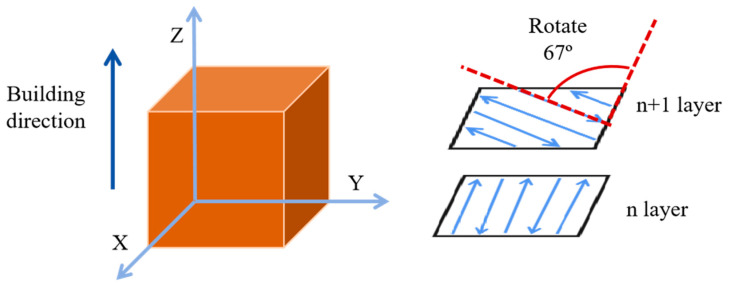
Schematics of a block specimen and the strip scanning strategy.

**Figure 3:**
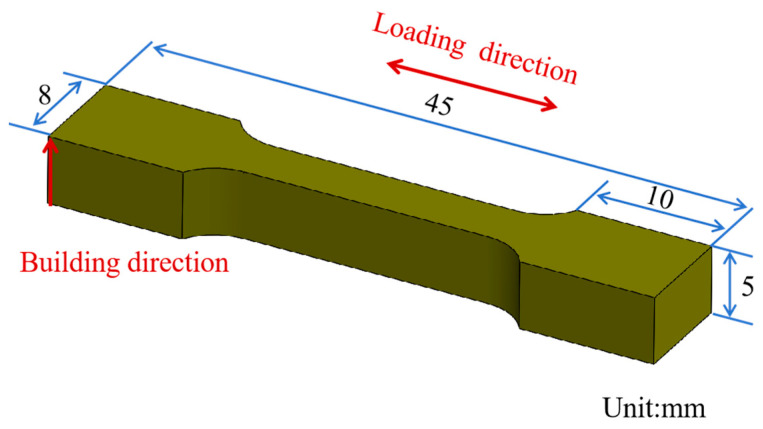
Schematic of tensile specimen.

The SLM125 3D printer from the German SLM Solution company (Lübeck, Germany) was used to prepare the specimens and was equipped with a single 400 W power IPG laser, which can achieve a maximum size of 125 × 125 × 125 mm^3^ to form the parts. The manufacturing process parameters that were selected are shown in Table 2, and a strip scanning strategy with an interlayer rotation of 67° was used, as shown in Figure 2. Block specimens with dimensions of 10 × 10 × 10 mm^3^ (Figure 2) and tensile specimens machined by wire-electrode cutting with reference standard ASTM/E8M [28] were used in this experiment, as shown in Figure 3.

### 2.2. Heat Treatment Process

The formed specimens were subjected to heat treatments at 500 °C (HT1), 800 °C (HT2), and 1100 °C (HT3) using the KF1200 box furnace supplied by the Nanjing Boyuntong Company (Nanjing, China), followed by air cooling after one hour of insulation, as shown in Table 3. After heat treatment, the specimens were polished with 2000-grit sandpaper to remove surface oxides in order to ensure accurate characterization.

### 2.3. Measurement and Characterization

The residual stresses of the specimens were determined using an X-ray residual stress analyzer (X-stress 3000, Vantaa, Finland). The X-ray tube used Mn, the diffraction angle was 154 °, Young’s modulus was 196 GPa, Poisson’s ratio was 0.28, Miller’s index was 311, the exposure time was 40 s, the tilt angle ranged from −45 ° to 45 °, the X-ray current was 7 mA, and the X-ray voltage was 30 kV. The phase composition of the specimens was analyzed using a Bruker AXS D8-02 X-ray diffractometer (Karlsruhe, Germany), where the Cu target was used as the radiation source and the scanning range was 20–120° with a scanning rate of 4°/min. The mechanically polished specimen was subjected to electrolytic polishing with a polishing voltage of 30 V and a polishing time of 10 s. The electrolyte was a mixture of HCLO_4_ and CH_3_CH_2_OH with a volume ratio of 2:8, and then the phase fraction was analyzed by an HKL Channel5 backscattering electron diffractometer (Oxfordshire, UK) with a test voltage of 30 kV, a scanning step of 1 μm, and an average confidence index of 0.33. Once the surface of the specimen was polished, the surface porosity was observed using an IE200M metallographic microscope(Sunny Optical Technology Group Co., Ltd., Zhejiang, China), and the pore area was calculated using ImageJ 1.52i to derive the densities of the specimens. The surface of the specimen was subjected to electrolytic polishing using a DPF-2 electrochemical etching instrument manufactured by Shanghai Metallurgical Machinery Co., Ltd. (Shanghai, China), and the polishing solution used was aqua regia (HCL:HNO_3_ = 3:1). The obtained microstructures were observed and analyzed at 5000× and 50,000× using an FEI Quanta 250 scanning electron microscope (Hillsboro, OR, USA) with an operating voltage of 20 kV. The tensile specimen model was prepared by sanding the surface of the powder adhesion with sandpaper to ensure a relatively smooth surface, thus avoiding surface defects during the tensile test. The U.S. MTS Exceed E45 tensile testing machine (Eden Prairie, MN, USA) was then used at room temperature, with a tensile rate of 0.008 mm/s, a tensile direction perpendicular to the PBF-LB/M scanning direction, and a data acquisition frequency of 30 Hz.

## 3. Experimental Results and Discussion

### 3.1. Microstructure

**Figure 4:**
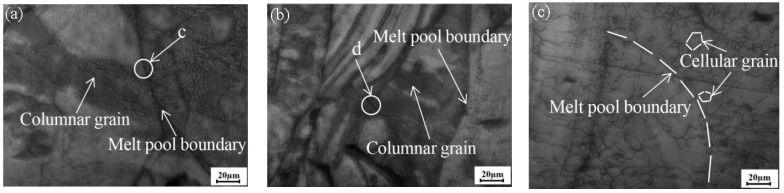

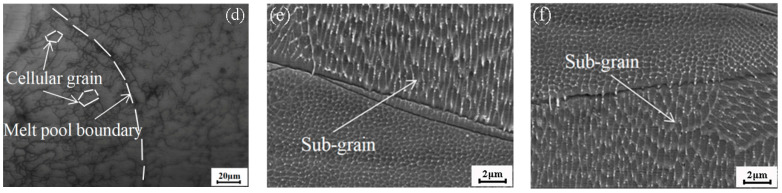
Microstructure of specimen with different heat treatments: (**a**) as-built; (**b**) HT1; (**c**) HT2; (**d**) HT3; (**e**) c area; (**f**) d area.

Figure 4a–d show the observed microstructures of the specimens in the initial and different heat-treated states of the subsequent cooling channel. In (a) and (e), it can be seen that the organization of the non-heat-treated specimen shows columnar grains; the growth direction runs along the melt pool boundary to the center of the melt pool; the length of which is more than 50 μm; and there is a dense distribution of subgrains with a diameter of about 0.51 μm in the interior of the specimen. The microstructure of specimens heat-treated at 500 °C did not show significant changes compared to the untreated samples (Figure 4b,f). After heat treatment at 800 °C and 1100 °C, the boundaries of the melt pool became blurred, the shape of the grains changed from columnar grains to cystalline grains, the diameter of the grains decreased to less than 20 μm, the subgranular boundaries inside the grains were dissolved, and the number of grain boundaries per unit area increased (Figure 4c,d).

In the unheated state, columnar grains were observed within the specimens and were accompanied by a dense distribution of subgrains within the grains, a finding that is consistent with previous observations [15,29]. No significant grain growth was observed in the PBF-LB/M-processed 316L SS specimens subjected to a 500 °C heat treatment. Due to the existence of subcrystalline structure within the as-built specimens of 316L SS, the effect of heat treatment on its microstructure is from the merger of subcrystalline boundaries and the gradual growth of subgrains [30]; 500 °C is much lower than the melting temperature of the 316L SS, so it did not produce changes in the microstructure. As the heat treatment temperature continues to increase to 800 °C and 1100 °C, which are two temperature conditions exceeding the recrystallization temperature threshold of stainless steel, the specimen undergoes a recrystallization reaction, resulting in grain size refinement. This transformation involves the conversion of columnar grains to cellular grains, which is accompanied by an increase in the number of grains. Concurrently, the subgrains undergo recrystallization at elevated temperatures, with recrystallization also occurring under dissolution. The microstructural alterations induced by distinct heat treatments exert a profound influence on the mechanical properties of the specimens, which will be elucidated in greater detail in the subsequent section.

### 3.2. Phase Analysis

**Figure 5:**
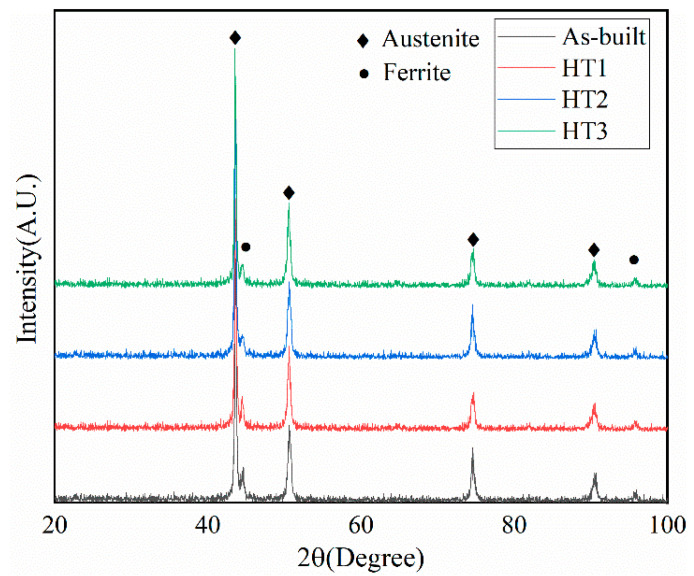
XRD patterns of 316L SS specimens under different heat treatment conditions.

**Figure 6:**
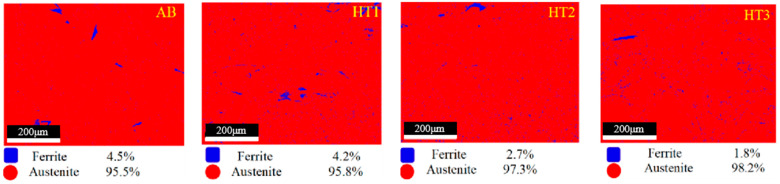
EBSD phase fractions of 316L SS specimens under different heat treatment conditions.

After analyzing the specimens using an XRD analyzer, it was found that they were mainly composed of the austenite phase in the various treatment states. However, the volume fraction of ferrite in the specimens in the 800 °C and 1100 °C heat-treated states was less than that of the specimens in the initial and 500 °C heat-treated states, as shown in Figure 5. The phase fractions of specimens were examined in detail by EBSD, and the results are presented in Figure 6. The ferrite volume fraction of the as-built specimen fabricated via PBF-LB/M was high at 4.5%, and there was no significant change in the ferrite volume fraction of the specimen that was heat-treated at 500 °C. The ferrite volume fraction of the specimens heat-treated at 800 °C and 1100 °C decreased significantly, and the extent of this decrease became more and more pronounced as the heat treatment temperature increased. The ferrite volume fraction of the specimens reached a minimum of 1.8% at the heat treatment temperature of 1100 °C.

In the PBF-LB/M process, the 316L SS material is first melted by the laser heat source, resulting in a liquid molten pool. Then, the molten pool enters the cooling process, where liquid stainless steel cooled to 1538 °C will crystallize, producing parts of high-temperature ferrite (δ phase), but liquid stainless steel still remains at this temperature. When the temperature drops to 1459 °C, the high-temperature ferrite and liquid stainless steel undergo an inclusion reaction to produce austenite. The inclusion reaction proceeds from the ferrite toward the center. Due to rapid cooling, some high-temperature ferrite becomes encapsulated by austenite before the transformation is fully completed, forming a massive residual δ ferrite.

### 3.3. Residual Stress

**Figure 7:**
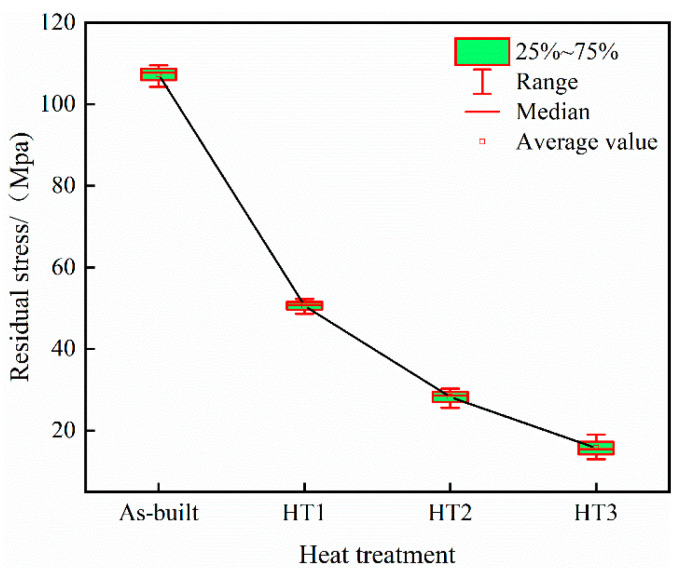
Variations in the residual tensile stress of specimens with different heat treatments.

Figure 7 depicts the trend for the residual tensile stress of PBF-LB/M specimens with different heat treatment methods, demonstrating that the residual tensile stress of the specimen in the as-built state is 107.6 MPa. After heat treatment at 500 °C, the residual tensile stress decreases to 50.65 MPa, representing a 53% reduction; at 800 °C, it further decreases to 28.7 MPa, corresponding to a 73% reduction; and when the heat treatment temperature reaches 1100 °C, it decreases to its lowest value of 15.5 MPa, representing an 85.59% reduction compared to the as-built specimen.

Due to the processing characteristics of “fast melting, fast cooling”, the internal thermal stress of the selected laser melting specimen is considerable. Stress Relief Annealing at 500 °C can eliminate approximately 50% of the residual stress, but this heat treatment temperature is low, which limits its impact on the specimen’s grain size and morphology. Therefore, the residual stress cannot be completely dissipated. As the heat treatment temperature is increased to 800 °C and 1100 °C, the specimen undergoes a recrystallization reaction, during which the grain is refined and the subgrain structure is dissolved. The dissolution of subgrains helps to reduce stress concentration within the material, which in turn reduces the residual stress of the specimen. Consequently, the PBF-LB/M specimens subjected to heat treatment at 1100 °C exhibit the lowest residual stress.

### 3.4. Relative Density

**Figure 8:**
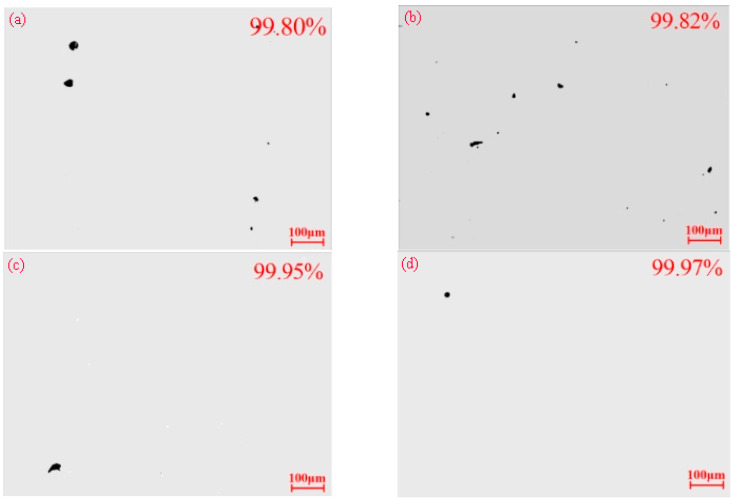
Pore observations of specimens with different heat treatments: (**a**) as-built; (**b**) HT1; (**c**) HT2; (**d**) HT3.

**Figure 9:**
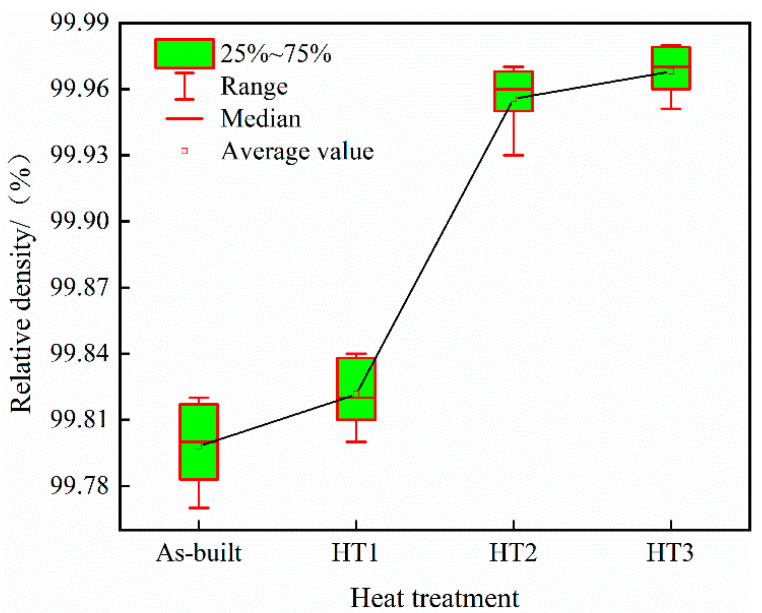
Variations in the relative densities of specimens with different heat treatments.

Figure 8 illustrates the surface porosity of each specimen, and Figure 9 depicts the trend in the densification of PBF-LB/M specimens subjected to different heat treatments. The relative density value of the specimen after heat treatment at 500 °C is 99.82%, which is not considerably different from that of the as-built state specimen. In contrast, the relative density of the specimen after heat treatment at 800 °C is 99.95%, and that after heat treatment at 1100 °C is 99.97%, which represents a notable improvement compared with that of the as-built state specimen.

Heat treatments can improve the powder’s metallurgical bond strength within parts fabricated via PBF-LB/M [22]. Higher heat treatment temperatures enhance metallurgical bonding, which in turn positively contributes to the increase in the relative density of the specimen. In addition, the finer microstructure can also improve the relative density of the specimen [26]. In this experiment, heat treatment at 500 °C did not significantly alter the grain structure of the specimens. Due to the limited effect of the lower temperature on enhancing metallurgical bonding, the relative density remained nearly unchanged compared to the as-built specimens. When the specimens are heat-treated at 800 °C and 1100 °C, recrystallization occurs, leading to grain refinement and a more uniform microstructure. Additionally, higher heat treatment temperatures enhance the metallurgical bonding strength of the metal powder, thereby significantly increasing the relative density of the specimens.

### 3.5. Mechanical Property

**Figure 10:**
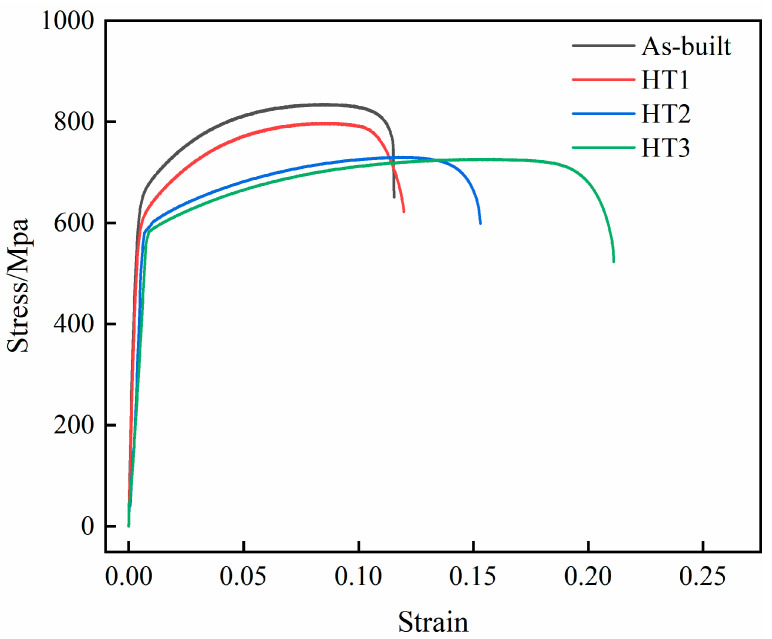
Stress–strain curves of tensile specimens with different heat treatments.

**Figure 11:**
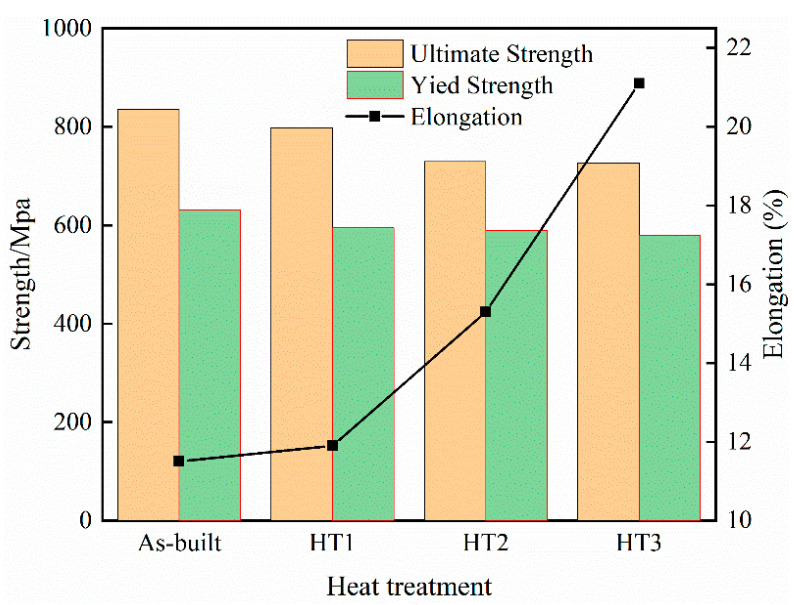
Variations in specimen strength and elongation with different heat treatments.

In Figure 10, the stress–strain curves of the as-built specimens fabricated via PBF-LB/M and specimens under different heat treatment temperatures are shown, and Figure 11 shows the trend graph lines of the strength properties and elongation of each specimen. The initial specimen exhibited a tensile strength of 835.2 MPa, a yield strength of 631.5 MPa, and an elongation of 11.5%. After heat treatment at 500 °C, the tensile and yield strengths slightly decreased to 797.6 MPa and 594.6 MPa, respectively, while elongation increased marginally to 11.9%, indicating a negligible change compared to the as-built condition. With further increases in heat treatment temperature, the strength properties declined more significantly, whereas elongation notably improved. Specifically, at 800 °C and 1100 °C, the specimen tensile strengths are 729.4 MPa and 726.3 MPa, the yield strengths are 589.52 MPa and 578.89 MPa, and the elongation values are 15.3% and 21.1%, respectively.

The 316L SS specimens exhibited high strength properties in the as-built and 500 °C heat-treated states, mainly due to the presence of dense subgranular boundaries and high-temperature ferrite strengthening phases [27]. As the heat treatment temperature increases to 800 °C and 1100 °C, the subgranular boundaries begin to dissolve, and the high-temperature ferrite strengthening phase undergoes a transformation, leading to a reduction in material strength. Although finer grain structures, increased density, and reduced residual stress may have a beneficial effect, their contribution is relatively minor compared to the influence of ferrite phase transformation. The elongation of the specimens is also influenced by a number of factors. On the one hand, the presence of subgrains provides a large area for coordinated material deformation, which is conducive to an increase in strain. On the other hand, the presence of high-temperature ferrite strengthening phases and residual stresses has a negative effect on material elongation. In the case studied, the presence of high-temperature ferrite strengthening phases and high residual stresses in the initial specimen grain are the main reasons for the low elongation, while the residual stresses in the specimen decrease under heat treatment at 500 °C, resulting in a slight increase in elongation. At heat treatment temperatures of 800 °C and 1100 °C, the high-temperature ferrite strengthening phase transforms, leading to a substantial reduction in residual stress and a notable increase in specimen elongation. Overall, the heat-treated specimens still have relatively satisfactory strength properties (YS > 550 MPa; UTS > 700 MPa), but the higher temperature heat treatment (800 °C and above) can significantly improve the plastic properties of the specimens. Therefore, heat treatment has a beneficial effect on the overall mechanical performance of the specimens, and the best combination of strength and elongation can be obtained at 1100 °C.

### 3.6. Comparison with Similar Results

Comprehensive current research on the heat treatment of additively manufactured 316L SS specimens found that heat treatment can dissolve the subgrain structure and make the microstructure more uniform, but it led to a decrease in the mechanical properties of the specimens. Due to the faster cooling rate of air and water cooling, grain coarsening occurs faster, and the decline in mechanical properties is larger [31]. However, the cooling rate of furnace cooling is lower, and its impact on mechanical properties is smaller, which is also an advantage of this study in choosing furnace cooling [32]. Currently, for the heat treatment of the 316L phase transition, the effect of densification is less, and the results of the study are different to previous results: some studies believe that heat treatment will not affect the densification of the specimens [23], but some are also consistent with the results of this study, finding that heat treatment can improve the densification [19]. At the same time, current heat treatment research with comparative analyses on residual stress has been relatively little explored. Slower cooling can reduce residual stress [19,23], but fast cooling may introduce stress and reduce the toughness of the material [17]. The relevant comparison is shown in Table 4. In summary, this study complements the current research results, demonstrating that the heat treatment process of the additive manufacturing of 316 SS has a certain reference value. In the future, further analyses will be carried out in follow-up studies to explore the impact of the relationship between the physical phase transition and residual stress under heat treatment conditions on the performance of 316L stainless steel for a more in-depth analysis.

## 4. Conclusions

In this study, the mechanism behind the effect of heat treatments at different temperatures on the microstructure, phase composition, residual stress, mechanical properties, and relative density of 316L SS samples fabricated using PBF-LB/M was investigated, and the following conclusions were drawn:

(1) The microstructure of the specimens formed via PBF-LB/M is columnar grains growing along the melt pool boundary, in which subgrains are distributed. High-temperature heat treatment (800 °C and above) dissolves subgrains in 316L SS specimens, resulting in refined and more uniform grain structures that contribute to residual stress reduction. Additionally, elevated temperatures enhance the metallurgical bonding strength between metal powders, thereby significantly increasing specimen densification. After the heat treatment at 1100 °C, the residual stress is reduced by 85.59% compared with the original component, and the density can reach up to 99.97%.
(2) The main composition of the 316L SS specimens manufactured via PBF-LB/M was austenite with a small amount of ferrite. Heat treatment at 500 °C did not result in a significant change in the composition of the specimens, whereas heat treatments at 800 °C and 1100 °C resulted in the transformation of the high-temperature ferrite phase formed during PBF-LB/M fabrication, with the ferrite content of the specimens reaching a minimum of 1.8% at a heat treatment of 1100 °C.
(3) Heat treatment led to a reduction in the strength of 316L SS specimens fabricated via PBF-LB/M, while elongation increased. This trend became more significant with increasing heat treatment temperature. Specifically, at 1100 °C, the tensile and yield strengths decreased by 13.03% and 8.33%, respectively, compared to the as-built specimens, while elongation markedly increased by 83.48%. These results indicate that high-temperature heat treatments have beneficial effects on the overall mechanical performance of the material.

## Figures and Tables

**Table 1:** Chemical composition of 316L stainless steel powder (mass).

Element	Fe	Cr	Ni	Mo	Mn	Si	C	P	S
Mass fraction (%)	68.635	16.92	10.63	2.46	0.74	0.59	0.011	0.01	0.004

**Table 2:** Manufacturing process parameters.

Laser Power (W)	Scanning Speed (mm/s)	Hatching Distance (μm)	Layer Thickness (μm)
190	750	100	30

**Table 3:** Design of a single-factor experiment of heat treatment.

Heat Treatment (HT)	HT Cycle
As-built	-
HT1	500 °C + 1 h + furnace cooling
HT2	800 °C + 1 h + furnace cooling
HT3	1100 °C + 1 h + furnace cooling

**Table 4:** Comparisons of studies with similar discussions and results.

No.	Cooling Methods	Microstructure	Phase Analysis	Residual Stress	Relative Density	Mechanical Property	Ref.
1	air cooling or water cooling	no grain enlargement, but the cellular dendritic structure is completely dissolved	N.A. ^1^	N.A.	N.A.	hardness and yield strength decrease	[17]
2	water quenching	more uniform	ferrite phase reduction	N.A.	N.A.	hardness decreases	[31]
3	furnace cooling or air cooling	subgrain structures and melting pool boundary dissolution	no comparative analysis	N.A.	N.A.	hardness and strength decrease; ductility increases	[32]
4	air cooling	more uniform	N.A.	decrease	increase	N.A.	[19]
5	air cooling	subgrain structures dissolution	no change	decrease	no change	strength decrease	[23]
6	furnace cooling	subgrain structures dissolution	ferrite phase reduction	decrease	increase	strength decrease	this work

^1^ Not studied in this work

## Data Availability

The original contributions presented in this study are included in the article. Further inquiries can be directed to the corresponding authors.

## References

[1] Navarro M., Michiardi A., Castaño O., Planell J.A. (2008). Biomaterials in Orthopaedics. J. R. Soc. Interface.

[2] D’Andrea D. (2023). Additive Manufacturing of AISI 316L Stainless Steel: A Review. Metals.

[3] Savolainen J., Collan M. (2020). How Additive Manufacturing Technology Changes Business Models?—Review of Literature. Addit. Manuf..

[4] Simoes M., Harris J.A., Ghouse S., Hooper P.A., McShane G.J. (2022). Process Parameter Sensitivity of the Energy Absorbing Properties of Additively Manufactured Metallic Cellular Materials. Mater. Des..

[5] Mercelis P., Kruth J. (2006). Residual Stresses in Selective Laser Sintering and Selective Laser Melting. Rapid Prototyp. J..

[6] Wang Z., Yang S., Huang Y., Fan C., Peng Z., Gao Z. (2021). Microstructure and Fatigue Damage of 316L Stainless Steel Manufactured by Selective Laser Melting (SLM). Materials.

[7] Peng T., Chen C. (2018). Influence of Energy Density on Energy Demand and Porosity of 316L Stainless Steel Fabricated by Selective Laser Melting. Int. J. Precis. Eng. Manuf. Green Technol..

[8] Yang D.C., Kan X.F., Gao P.F., Zhao Y., Yin Y.J., Zhao Z.Z., Sun J.Q. (2022). Influence of Porosity on Mechanical and Corrosion Properties of SLM 316L Stainless Steel. Appl. Phys. A.

[9] Röttger A., Boes J., Theisen W., Thiele M., Esen C., Edelmann A., Hellmann R. (2020). Microstructure and Mechanical Properties of 316L Austenitic Stainless Steel Processed by Different SLM Devices. Int. J. Adv. Manuf. Technol..

[10] Jiang H.Z., Li Z.Y., Feng T., Wu P.Y., Chen Q.S., Feng Y.L., Chen L.F., Hou J.Y., Xu H.J. (2021). Effect of Process Parameters on Defects, Melt Pool Shape, Microstructure, and Tensile Behavior of 316L Stainless Steel Produced by Selective Laser Melting. Acta Metall. Sin. Engl. Lett..

[11] Antony K., Arivazhagan N., Senthilkumaran K. (2014). Numerical and Experimental Investigations on Laser Melting of Stainless Steel 316L Metal Powders. J. Manuf. Processes.

[12] Deng Y., Mao Z., Yang N., Niu X., Lu X. (2020). Collaborative Optimization of Density and Surface Roughness of 316L Stainless Steel in Selective Laser Melting. Materials.

[13] Liu Y., Zhang M., Shi W., Ma Y., Yang J. (2021). Study on Performance Optimization of 316L Stainless Steel Parts by High-Efficiency Selective Laser Melting. Opt. Laser Technol..

[14] Wang D., Wu S., Yang Y., Dou W., Deng S., Wang Z., Li S. (2018). The Effect of a Scanning Strategy on the Residual Stress of 316L Steel Parts Fabricated by Selective Laser Melting (SLM). Materials.

[15] Shin W.S., Son B., Song W., Sohn H., Jang H., Kim Y.J., Park C. (2021). Heat Treatment Effect on the Microstructure, Mechanical Properties, and Wear Behaviors of Stainless Steel 316L Prepared via Selective Laser Melting. Mater. Sci. Eng. A.

[16] Kamariah M.S.I.N., Harun W.S.W., Khalil N.Z., Ahmad F., Ismail M.H., Sharif S. (2017). Effect of Heat Treatment on Mechanical Properties and Microstructure of Selective Laser Melting 316L Stainless Steel. IOP Conf. Ser. Mater. Sci. Eng..

[17] Sistiaga M.L.M., Nardone S., Hautfenne C., Humbeeck J.V. Effect of Heat Treatment of 316L Stainless Steel Produced by Selective Laser Melting (SLM). Proceedings of the 27th Annual International Solid Freeform Fabrication Symposium—An Additive Manufacturing Conference.

[18] Zhou Y., Duan L., Li F., Chen K., Wen S. (2022). Effect of Heat Treatment on the Microstructure and Mechanical Property of W/316L Multi-Material Fabricated by Selective Laser Melting. J. Alloys Compd..

[19] Tascioglu E., Karabulut Y., Kaynak Y. (2020). Influence of Heat Treatment Temperature on the Microstructural, Mechanical, and Wear Behavior of 316L Stainless Steel Fabricated by Laser Powder Bed Additive Manufacturing. Int. J. Adv. Manuf. Technol..

[20] Morozova I., Kehm C., Obrosov A., Yang Y., Miah K.U.M., Uludintceva E., Fritzsche S., Weiß S., Michailov V. (2023). On the Heat Treatment of Selective-Laser-Melted 316L. J. Mater. Eng. Perform..

[21] Ronneberg T., Davies C.M., Hooper P.A. (2020). Revealing Relationships between Porosity, Microstructure and Mechanical Properties of Laser Powder Bed Fusion 316L Stainless Steel through Heat Treatment. Mater. Des..

[22] Kurzynowski T., Gruber K., Stopyra W., Kuźnicka B., Chlebus E. (2018). Correlation between Process Parameters, Microstructure and Properties of 316 L Stainless Steel Processed by Selective Laser Melting. Mater. Sci. Eng. A.

[23] Chao Q., Thomas S., Birbilis N., Cizek P., Hodgson P.D., Fabijanic D. (2021). The Effect of Post-Processing Heat Treatment on the Microstructure, Residual Stress and Mechanical Properties of Selective Laser Melted 316L Stainless Steel. Mater. Sci. Eng. A.

[24] Kong D.C., Ni X.Q., Dong C.F., Zhang L., Man C., Yao J.Z., Xiao K., Li X.G. (2018). Heat Treatment Effect on the Microstructure and Corrosion Behavior of 316L Stainless Steel Fabricated by Selective Laser Melting for Proton Exchange Membrane Fuel Cells. Electrochim. Acta.

[25] Xiao Q., Chen J.J., Lee H.B., Jang C., Jang K. (2023). Effect of Heat Treatment on Corrosion Behaviour of Additively Manufactured 316L Stainless Steel in High-Temperature Water. Corros. Sci..

[26] Liu W., Liu C.S., Wang Y., Zhang H., Ni H.W. (2024). Effect of Heat Treatment on the Corrosion Resistance of 316L Stainless Steel Manufactured by Laser Powder Bed Fusion. J. Mater. Res. Technol..

[27] Laleh M., Hughes A.E., Xu W., Cizek P., Tan M.Y. (2020). Unanticipated Drastic Decline in Pitting Corrosion Resistance of Additively Manufactured 316L Stainless Steel after High-Temperature Post-Processing. Corros. Sci..

[28] (2024). Standard Test Methods for Tension Testing of Metallic Materials.

[29] Saeidi K., Gao X., Zhong Y., Shen Z.J. (2015). Hardened Austenite Steel with Columnar Sub-Grain Structure Formed by Laser Melting. Mater. Sci. Eng. A.

[30] Saeidi K., Akhtar F. (2018). Subgrain-Controlled Grain Growth in the Laser-Melted 316 L Promoting Strength at High Temperatures. R. Soc. Open Sci..

[31] Benarji K., Ravi Kumar Y., Jinoop A.N., Paul C.P., Bindra K.S. (2021). Effect of Heat-Treatment on the Microstructure, Mechanical Properties and Corrosion Behaviour of SS 316 Structures Built by Laser Directed Energy Deposition Based Additive Manufacturing. Met. Mater. Int..

[32] Waqar S., Liu J.W., Sun Q.D., Guo K., Sun J. (2020). Effect of Post-Heat Treatment Cooling on Microstructure and Mechanical Properties of Selective Laser Melting Manufactured Austenitic 316L Stainless Steel. Rapid Prototyp. J..

